# Temporal effects of exercise on retrieval-induced forgetting

**DOI:** 10.1007/s00426-026-02355-w

**Published:** 2026-08-11

**Authors:** Paul D. Loprinzi, David O. Shopeju, Benjamin C. Storm

**Affiliations:** 1 Exercise & Memory Laboratory, Department of Kinesiology and Sport, The University of Mississippi, 229 Turner Center, Oxford, MS 38677 United States; 2 UCSC Memory Laboratory, Department of Psychology, University of California Santa Cruz, Santa Cruz, CA 95064 United States

## Abstract

Retrieval-induced forgetting (RIF) refers to the phenomenon whereby selectively retrieving some information impairs later recall of related, non-retrieved information, often interpreted as reflecting inhibitory control processes during memory retrieval. Recent work suggested that acute aerobic exercise performed between repeated memory tests may preserve the RIF effect over time. The present study sought to directly replicate and extend this finding using a larger and more controlled experimental design. Healthy young adults (*N* = 372) completed a standard category-exemplar RIF task involving selective retrieval practice followed by two final test assessments. Between the two tests, participants were randomly assigned to one of five conditions that manipulated exercise exposure, duration, and order (exercise, standing control, or combined sequences). Exercise was performed on a treadmill at vigorous intensity (80% heart-rate reserve), whereas control conditions involved standing on a stationary treadmill. Replicating the classic RIF effect, recall for Rp- items was significantly lower than for Nrp- items. However, contrary to prior findings, acute exercise did not preserve RIF across the two tests. Instead, the magnitude of RIF decreased from the first to the second test across all groups, driven primarily by improved recall of previously suppressed items. These findings indicate that RIF can attenuate with repeated retrieval but fail to provide evidence that acute exercise acts to stabilize retrieval-induced forgetting.

## Introduction

Retrieval-induced forgetting (RIF) refers to the phenomenon whereby selectively retrieving some previously learned information impairs subsequent recall of related, non-retrieved information (Anderson et al., 1994). The phenomenon is typically investigated using a category-exemplar paradigm in which participants first study multiple exemplars belonging to several semantic categories (e.g., FRUIT–orange, METAL-silver, FRUIT–banana, METAL–copper). During a subsequent retrieval-practice phase, participants repeatedly retrieve a subset of exemplars from a subset of categories (e.g., FRUIT–or___), while the remaining exemplars receive no retrieval practice. At final test, memory is assessed for four classes of items: practiced exemplars from practiced categories (Rp+), unpracticed exemplars from practiced categories (Rp−), baseline exemplars from unpracticed categories corresponding to practiced items (Nrp+), and baseline exemplars from unpracticed categories corresponding to unpracticed items (Nrp−). The defining characteristic of RIF is that Rp− items are recalled less well than Nrp− items, suggesting that selective retrieval practice acts to reduce the accessibility of non-practiced exemplars associated with practiced categories.

The empirical observation of RIF has been replicated across numerous experimental paradigms and has generally been interpreted as reflecting the consequences of adaptive inhibitory control processes that facilitate efficient memory retrieval by resolving competition among related representations (Anderson, 2003; Murayama et al., 2014; Storm et al., 2015; Storm & Levy, 2012). Specifically, Rp- items are assumed to be inhibited during retrieval practice to facilitate access to Rp+ items. As a consequence of this inhibition, however, when they are subsequently tested themselves, they are impaired relative to baseline. Alternative accounts have argued that inhibition is not necessary to account for RIF (e.g., Jonker et al., 2013; Raaijmakers & Jakab, 2013; Verde, 2012). According to the context account, for example, Jonker and colleagues have argued that retrieval practice induces a change in mental context between study and final test, and it is this change in context that makes it difficult to retrieve Rp- items because participants are prompted to search the retrieval-practice context for Rp- items even though such items are not actually associated with that context, thus leading to RIF.

RIF has been shown to be highly robust across materials, paradigms, and populations (Murayama et al., 2014; Storm et al., 2015). However, its magnitude is sensitive to contextual and cognitive factors, including working memory capacity and affective state (e.g., Aslan & Bäuml, 2011; Bäuml & Kuhbandner, 2007). Of relevance to the present investigation, RIF has been observed to attenuate across repeated tests and longer delays (e.g., a 24-hr or 1-week delay), often through increased recall of previously suppressed (Rp-) items, a pattern that has been attributed to the transient nature of the inhibitory effect and the strengthening of suppressed representations over time (MacLeod & Macrae, 2001; Storm et al., 2012).

Recent research has begun to explore whether acute physical exercise modulates RIF (Loprinzi & Storm, 2023). Acute exercise reliably enhances aspects of episodic memory, executive control, and interference resolution, potentially through arousal-related neuromodulatory mechanisms involving catecholamines, neurotrophic factors, and prefrontal engagement (Chang et al., 2012; Loprinzi et al., 2017; Oberste et al., 2019). Because inhibitory control is central to prominent theoretical accounts of RIF (Storm & Levy, 2012), acute exercise has been proposed as a candidate manipulation for influencing forgetting as well as remembering.

In a recent series of experiments, Loprinzi and Storm (2023) evaluated the effects of acute treadmill exercise on RIF. Across three experiments, exercise prior to encoding did not influence RIF. However, Experiment 3 demonstrated that when exercise occurred between two cued-recall assessments undertaken in relatively short succession (25-min delay), the RIF effect was preserved, whereas RIF diminished in a seated control condition. In other words, exercise appeared to prevent the reduction in RIF observed between two final tests.

Despite its theoretical importance, the mechanisms underlying this preservation effect remain unresolved. One possibility is that acute exercise helps stabilize the inhibitory process established during retrieval practice, such as by increasing catecholaminergic activity, cerebral perfusion, and prefrontal cortical activation (Chang et al., 2012; McMorris et al., 2008). Under this account, acute aerobic exercise may prolong the suppression of competing memory representations, thereby maintaining RIF across repeated assessments.

An alternative explanation, derived from the context-based account of RIF (Jonker et al., 2013), is that acute exercise may influence the retrieval strategies participants employ on the second test. One reason RIF effects may diminish over time is that participants may become less likely to search for Rp- items in the retrieval-practice context, thus circumventing the context-cueing effects argued to cause RIF. Acute exercise and its associated arousal could affect retrieval strategies by leading participants to rely on the most dominant, highly accessible context, thus leading them to continue to search the inappropriate retrieval-practice context for Rp- items instead of the study phase. As such, exercise could effectively reinstate the competitive dynamics that drive RIF, thus preventing the recovery of Rp- items.

Beyond these exercise-specific physiological mechanisms, the prior preservation effect may alternatively reflect the limitations of the seated baseline used in previous work. Because Experiment 3 of Loprinzi and Storm (2023) compared treadmill exercise to a seated rest control, the apparent preservation of RIF in the exercise group may have actually reflected accelerated attenuation in the control group. Sitting quietly for an extended time induces relaxation, lowers baseline physiological arousal, and creates a shift in physical context. This drop in attention or engagement may have caused participants to drift out of the retrieval-practice context, hastening the reduction of the RIF effect. Said differently, simply remaining upright and alert may have been sufficient to prevent contextual drift, independent of any cardiovascular exertion. To isolate the true effects of acute exercise from these postural and environmental confounds, the present study employed a more rigorous standing control.

Furthermore, any apparent preservation of RIF must be disentangled from the natural temporal evolution of RIF itself, which tends to diminish over time across successive assessments. Presumably, a delay between two final tests may allow Rp- items to recover from either inhibition or whatever context- or interference-based mechanism may have been impeding the recall of those items. To distinguish among competing explanations, the present study systematically manipulated intervention type, intervention duration, temporal proximity of exercise to the first and second final-test assessments, and the interval separating those assessments. By independently varying these factors across five experimental conditions (including rigorous standing controls), the present design provides a more comprehensive examination of the mechanisms governing the temporal evolution of RIF than was possible in the original experiment.

Indeed, the explanations discussed above yield distinct predictions regarding the timing of exercise during the delay interval. If RIF is preserved owing to the acute physiological effects of exercise associated with enhanced executive control, increased prefrontal cortical activation, etc., then exercise immediately following the first test should be relatively more likely to sustain the inhibitory effects observed on that first test. In contrast, from the perspective of the context-based account, exercise occurring immediately before the second test should be more likely to maintain RIF on that second test. Specifically, if exercise promotes reliance on the retrieval-practice context, participants should continue searching that inappropriate context when attempting to retrieve Rp- items, thereby preserving the context-based forgetting effect rather than allowing the typical attenuation of RIF across repeated tests. By systematically manipulating the temporal proximity of exercise to the first versus the second final test, the present design provides not only a replication of the preservation effect observed by Loprinzi and Storm (2023), but a test between these theoretical accounts to determine whether exercise modifies the natural temporal evolution of inhibitory or context-based retrieval processes.

## Methods

### Participants

Participants were healthy young adults recruited from a university population via classroom announcements and word of mouth. Inclusion and exclusion criteria matched those used by Loprinzi and Storm (2023). Participants were excluded if they reported recent concussion or head injury, neurological or psychiatric disorders, pregnancy, daily smoking, recent substance use, or failure to meet exercise safety guidelines. Participants were instructed to refrain from caffeine and vigorous exercise prior to testing.

A total of 372 participants completed the study and were randomly assigned to one of the five experimental conditions. This sample size substantially exceeded that of Experiment 3 in Loprinzi and Storm (2023), which included 158 participants. To evaluate the sensitivity of the present design, we conducted a sensitivity power analysis for the primary mixed repeated-measures interaction examined in the study. Assuming α = 0.05, power = 0.80, five between-subjects groups, two repeated assessments, two item types, and an assumed repeated-measures correlation of 0.50, the present sample provided adequate power to detect a small interaction effect (Cohen’s *f* = 0.09). Thus, the current study was substantially better powered than the original experiment to detect exercise-related modulation of RIF. Although extremely small interaction effects cannot be excluded, the present sample provides a more precise estimate of the likely magnitude of any exercise-related preservation effect.

All participants provided written informed consent. The study was approved by the institutional review board and conducted in accordance with the Declaration of Helsinki.

### Design

**Fig. 1:**
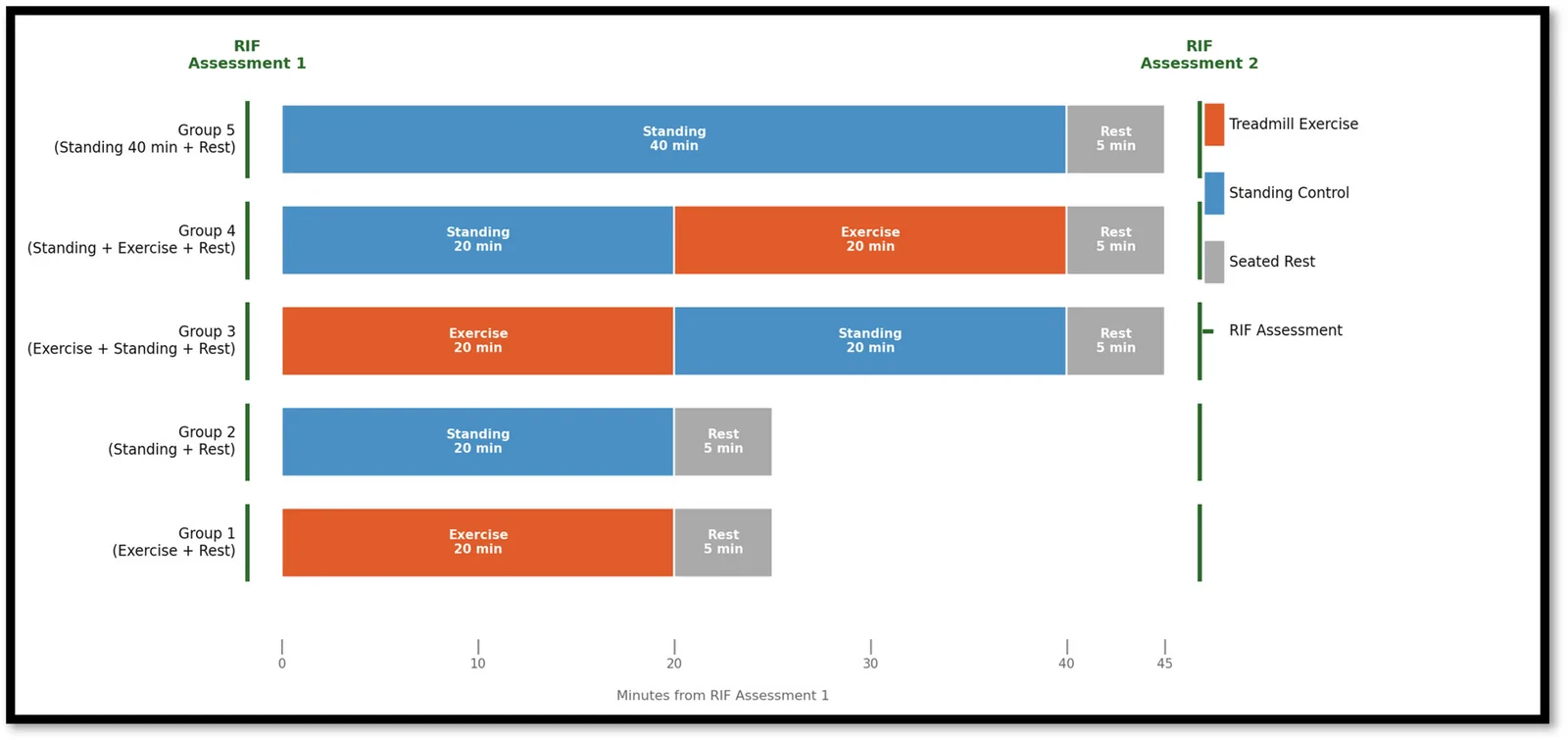
Schematic of the five experimental conditions between Test 1 and Test 2. Exercise periods are shown in orange-red, standing control periods in blue, and seated rest in gray. Groups 1 and 2 completed a 25-minute interval; Groups 3, 4, and 5 completed a 45-minute interval. Temporal proximity of exercise to the second assessment differed across conditions

The experiment employed a mixed factorial design with Group (five levels) as a between-subjects factor and Assessment (Test 1 vs. Test 2) and Item Type (Rp+, Rp-, Nrp+, Nrp-) as within-subjects factors (Fig. 1).

Between the two RIF assessments, participants completed one of the following conditions:

1. Exercise + Rest: 20 min treadmill exercise + 5 min seated rest.
2. Standing Control + Rest: 20 min treadmill standing + 5 min seated rest.
3. Exercise + Standing + Rest: 20 min exercise + 20 min standing + 5 min rest.
4. Standing + Exercise + Rest: 20 min standing + 20 min exercise + 5 min rest.
5. Standing Control (40 min) + Rest: 40 min standing + 5 min rest.

Participants were randomly assigned to groups. All testing occurred individually in a quiet laboratory environment.

### Retrieval-induced forgetting task

The RIF task followed the standard category-exemplar paradigm originally developed by Anderson et al. (1994). Participants first studied category-exemplar pairs (e.g., FRUIT–orange, FRUIT–banana, FRUIT–grape, ANIMAL–tiger, ANIMAL–lion). During the subsequent retrieval-practice phase, participants selectively retrieved only a subset of exemplars from a subset of categories by completing category-plus-stem cues (e.g., FRUIT – or___, with the correct response being orange). Importantly, only selected exemplars received retrieval practice, whereas related exemplars from the same category received no retrieval practice.

Four classes of items were subsequently evaluated. Rp+ items were practiced exemplars from practiced categories (e.g., orange). Rp− items were unpracticed exemplars from practiced categories (e.g., banana), which served as the critical measure of RIF because they competed with practiced items during selective retrieval but were themselves never practiced. Nrp+ items were baseline low-frequency exemplars from categories that did not receive retrieval practice, providing an appropriate comparison for Rp+ items. Nrp− items were baseline high-frequency exemplars from unpracticed categories and served as the comparison condition for Rp− items. Retrieval-induced forgetting is operationally defined as lower recall of Rp− items relative to Nrp− items, reflecting reduced accessibility of nonretrieved competitors following selective retrieval practice.

Consistent with Experiment 3 of Loprinzi and Storm (2023), each semantic category contained both high-frequency and low-frequency exemplars. High-frequency exemplars always served as Rp − or Nrp− items, whereas low-frequency exemplars served as Rp + or Nrp+ items. This arrangement follows standard RIF methodology and was intentionally adopted because high-frequency category exemplars are presumed to generate greater competition during selective retrieval practice, thereby increasing the likelihood that inhibitory processes are recruited (Anderson et al., 1994). Consequently, baseline recall levels differ between the high-frequency and low-frequency item sets by design and should not be interpreted as reflecting experimental item effects.

### Exercise and control protocols

Exercise protocols matched those used by Loprinzi and Storm (2023). Participants exercised on a motorized treadmill at vigorous intensity corresponding to an age-predicted target heart-rate range (80% of heart rate reserve). Heart rate (Polar H10) was monitored throughout. Standing control conditions involved standing upright on a stationary treadmill at 0 mph. This control was selected to equate posture, environmental exposure, and expectancy while minimizing cardiovascular engagement.

### Analyses

Our aim was to evaluate if exercise influenced RIF. To investigate this aim, a 5 (group: 1, 2, 3, 4, 5) × 2 (item: Rp − vs. Nrp−) × 2 (test: 1 vs. 2) repeated measures ANOVA was conducted to examine RIF. Similarly, a 5 (group: 1, 2, 3, 4, 5) × 2 (item: Rp + vs. Nrp+) × 2 (test: 1 vs. 2) repeated measures ANOVA was conducted to examine the retrieval practice effect. All analyses were computed in JASP (0.95.4). Huynh-Feldt correction was employed when sphericity was violated, and Bonferroni correction was used when evaluating post-hoc tests from the repeated-measures ANOVA.

## Results

### Participant characteristics

Table 1 displays demographic and behavioral characteristics. Groups were similar (all ps > 0.05) across all parameters.

**Table 1:** Demographic and behavioral characteristics

Variable	Group 1	Group 2	Group 3	Group 4	Group 5
N	75	75	77	71	74
Age (years)	20.1 (1.4)	20.1 (1.4)	20.2 (1.5)	20.5 (1.7)	20.2 (1.2)
Gender (% women)	64.0	68.0	58.4	62.0	67.6
BMI	24.1 (4.0)	24.4 (5.3)	24.5 (4.1)	24.5 (5.2)	24.4 (4.9)
MVPA (min/wk)	232.7 (192.5)	186.1 (145.4)	267.3 (333.5)	196.4 (171.2)	205.7 (153.0)

the following group manipulations occurred between the two RIF assessments

Group 1: Exercise + Rest: 20 min treadmill exercise + 5 min seated rest

Group 2: Standing Control + Rest: 20 min treadmill standing + 5 min seated rest

Group 3: Exercise + Standing + Rest: 20 min exercise + 20 min standing + 5 min rest

Group 4: Standing + Exercise + Rest: 20 min standing + 20 min exercise + 5 min rest

Group 5: Standing Control (40 min) + Rest: 40 min standing + 5 min rest

### Exercise intensity

Table 2 displays mean heart rate (bpm) recorded at each time point across the five groups. As expected, exercise groups (Groups 1, 3, and 4 during their respective exercise periods) achieved substantially elevated heart rates consistent with vigorous-intensity exercise, while control groups (Groups 2 and 5) maintained heart rates near resting levels throughout.

**Table 2:** Heart rate (bpm) across time period and group

Time period (min)	Group 1	Group 2	Group 3	Group 4	Group 5
Rest	79.4 (13.6)	79.2 (10.0)	79.3 (14.2)	81.1 (14.7)	81.2 (12.8)
5	149.1 (25.9)	82.5 (12.2)	147.7 (26.2)	86.0 (14.6)	83.3 (12.3)
10	165.0 (17.1)	82.6 (12.3)	158.9 (23.4)	86.9 (14.1)	84.8 (13.2)
15	168.7 (13.7)	82.8 (12.5)	162.6 (22.9)	88.3 (15.8)	83.8 (12.5)
20	171.8 (13.0)	82.5 (12.8)	162.1 (23.1)	98.5 (28.6)	83.9 (13.4)
25	104.8 (13.3)	75.7 (12.3)	115.6 (17.8)	151.2 (20.6)	85.4 (11.7)
30	-	-	109.8 (18.1)	163.9 (17.1)	85.4 (12.4)
35	-	-	107.2 (18.2)	168.7 (14.6)	84.5 (12.0)
40	-	-	103.2 (18.5)	169.2 (12.5)	83.8 (12.4)
45	-	-	90.1 (17.0)	102.8 (13.1)	74.1 (14.5)

Values are means (SD). Dashes indicate time points not applicable to the group’s condition. Groups 1 and 3 exercised during the first 20 min; Group 4 exercised during minutes 21–40; Groups 2 and 5 stood throughout

### Retrieval practice and retrieval-induced forgetting

Table 3 displays recall performance for all item types.

**Table 3:** Memory performance across group and item type

	RP		RIF		RIF Effect
	Rp+	Nrp+	Rp-	Nrp-	Rp- − Nrp-
First Test					
Group 1 (*n* = 75)	0.514 (0.16)	0.231 (0.14)	0.354 (0.14)	0.431 (0.14)	− 0.077
Group 2 (*n* = 75)	0.524 (0.16)	0.238 (0.15)	0.358 (0.14)	0.431 (0.16)	− 0.073
Group 3 (*n* = 77)	0.500 (0.17)	0.223 (0.13)	0.363 (0.13)	0.429 (0.17)	− 0.066
Group 4 (*n* = 71)	0.551 (0.15)	0.236 (0.13)	0.357 (0.14)	0.408 (0.15)	− 0.051
Group 5 (*n* = 74)	0.545 (0.18)	0.253 (0.13)	0.363 (0.13)	0.435 (0.14)	− 0.072
Second Test					
Group 1 (*n* = 75)	0.521 (0.16)	0.257 (0.15)	0.423 (0.14)	0.472 (0.13)	− 0.049
Group 2 (*n* = 75)	0.515 (0.17)	0.247 (0.16)	0.419 (0.16)	0.449 (0.17)	− 0.030
Group 3 (*n* = 77)	0.486 (0.17)	0.251 (0.14)	0.409 (0.14)	0.455 (0.17)	− 0.046
Group 4 (*n* = 71)	0.527 (0.15)	0.265 (0.14)	0.411 (0.15)	0.458 (0.14)	− 0.047
Group 5 (*n* = 74)	0.533 (0.18)	0.264 (0.14)	0.409 (0.13)	0.463 (0.14)	− 0.054

The following group manipulations occurred between the two RIF assessments

Group 1: Exercise + Rest: 20 min treadmill exercise + 5 min seated rest

Group 2: Standing Control + Rest: 20 min treadmill standing + 5 min seated rest

Group 3: Exercise + Standing + Rest: 20 min exercise + 20 min standing + 5 min rest

Group 4: Standing + Exercise + Rest: 20 min standing + 20 min exercise + 5 min rest

Group 5: Standing Control (40 min) + Rest: 40 min standing + 5 min rest

### Retrieval practice effect (Rp + vs. Nrp+)

A 5 (group: 1, 2, 3, 4, 5) × 2 (item: Rp + vs. Nrp+) × 2 (test: 1 vs. 2) repeated measures ANOVA was conducted to examine the retrieval practice effect. There was a significant main effect of item, *F*(1, 367) = 1262.26, *p* < .001, η² = 0.443, indicating that Rp+ items (*M* = 0.522, *SE* = 0.008) were recalled significantly better than Nrp+ items (*M* = 0.245, SE = 0.008). There was also a significant item × test interaction, *F*(1, 367) = 28.74, *p* < .001, η² = 0.001, suggesting that the retrieval practice benefit changed across the two tests. However, there was no significant main effect of group, *F*(4, 367) = 0.81, *p* = .521, η² = 0.003, no significant item × group interaction, *F*(4, 367) = 0.50, *p* = .736, η² < 0.001, no significant test × group interaction, *F*(4, 367) = 1.20, *p* = .310, η² < 0.001, and no significant three-way item × test × group interaction, *F*(4, 367) = 1.31, *p* = .266, η² < 0.001.

### Retrieval-induced forgetting (Rp − vs. Nrp−)

A 5 (group: 1, 2, 3, 4, 5) × 2 (item: Rp − vs. Nrp−) × 2 (test: 1 vs. 2) repeated measures ANOVA was conducted to examine RIF. There was a significant main effect of item, *F*(1, 367) = 54.54, *p* < .001, η² = 0.035, indicating that Rp- items (*M* = 0.382, SE = 0.007) were recalled significantly worse than Nrp- items (*M* = 0.441, SE = 007), demonstrating a RIF effect. There was also a significant main effect of test, *F*(1, 367) = 140.20, *p* < .001, η² = 0.021, showing that overall recall improved from the first test (*M* = 0.387, SE = 0.006) to the second test (*M* = 0.436, SE = 0.006). Additionally, there was a significant item × test interaction, *F*(1, 367) = 12.27, *p* < .001, η² = 0.001, indicating that the magnitude of the RIF effect changed across the two tests. Planned follow-up *t*-tests, collapsed across the five experimental groups because no significant interactions involving group were observed, confirmed that a significant RIF effect was present at both Test 1 (*RIF = − 0.069*, *t = -7.95*, *p* < .001) and Test 2 (*RIF = − 0.046*,* t = -5.81*, *p* < .001), though the magnitude of the effect was smaller on the second test. Further analysis revealed that this attenuation was driven by a significant increase in the recall of Rp- items from Test 1 to Test 2 (*0.055*,* t = 11.33*, *p* < .001) compared to a relatively smaller increase in the recall of Nrp- items from Test 1 to Test 2 (*0.032*,* t = 6.39*, *p* < .001).

Critically, there was no significant main effect of group, *F*(4, 367) = 0.10, *p* = .984, η² < 0.001, no significant item × group interaction, *F*(4, 367) = 0.15, *p* = .963, η² < 0.001, no significant test × group interaction, *F*(4, 367) = 1.18, *p* = .320, η² < 0.001, and no significant three-way item × test × group interaction, *F*(4, 367) = 1.04, *p* = .384, η² < 0.001. These null interactions indicate that the exercise manipulation did not differentially affect the RIF effect across groups or across the two test occasions.^1^

## Discussion

The present study was designed as a direct replication and extension of Experiment 3 from Loprinzi and Storm (2023), which previously demonstrated that acute exercise positioned between two final tests preserved the RIF effect across those assessments. Contrary to this prior finding, we did not replicate the exercise-related preservation of RIF. Instead, RIF was attenuated across all five experimental conditions, including those involving acute exercise. This outcome constitutes a failed replication of Experiment 3, but one that yields important theoretical and empirical insights into the dynamics of forgetting, repeated retrieval, and exercise.

Indeed, not only did acute exercise fail to affect RIF across repeated assessments, but it also failed to affect the benefits of retrieval practice. Specifically, no exercise-related interactions were observed for the comparison of Rp + and Nrp+ items, indicating that the benefits of retrieval practice were comparable across experimental conditions. Thus, the persistence of both retrieval-practice effects and RIF across repeated tests were similarly unaffected by exercise. Collectively, these findings suggest that acute exercise may not broadly impact the continuing consequences of retrieval in either direction, at least under the present conditions.

### Attenuation of RIF across repeated final test assessments

The magnitude of the RIF effect, operationalized as the difference between recall of Nrp − and Rp− items, was smaller on the second test than on the first across all experimental conditions. Specifically, the RIF effect decreased by approximately 0.4 to 4.3% points, reflecting greater improvement in recall of previously suppressed Rp− items than of baseline Nrp− items. This pattern is generally consistent with previous demonstrations that RIF can diminish across delays (MacLeod & Macrae, 2001; Storm et al., 2012), suggesting that RIF reflects a temporary retrieval-based phenomenon rather than a permanent reduction in memory accessibility. The present findings extend this literature by demonstrating that attenuation of RIF is evident even across relatively brief intervals of approximately 25 and 45 min separating successive assessments. Most previous work has examined attenuation across much longer delays such as days and weeks.

Several mechanisms may account for this attenuation. According to the prototypical inhibitory account of RIF, selective retrieval temporarily reduces the accessibility of competing representations, but these representations may subsequently recover as the inhibition responsible for impairing the recall of Rp- items is lifted (Anderson, 2003; Storm & Levy, 2012). Alternatively, from the perspective of non-inhibitory accounts, Rp- items may recover relative to Nrp items as retrieval competition diminishes or contextual cues change over time. As suggested by Estes (1955), the stimulus conditions supporting retrieval may evolve across successive tests, and an item that is not accessible on a first test may become accessible on a second test. In contrast, owing to the benefits of retrieval practice, most items successfully recalled on a first test are likely to remain recallable on a second test (Roediger & Karpicke, 2006). Rp- items may therefore be more likely to recover than Nrp- items for multiple reasons. First, the factors preventing their initial recall may dissipate over time. Second, as has been shown in prior work on hypermnesia (Payne, 1987), if they are successfully recalled despite their relatively impaired state, they are likely to remain recallable when tested again after a delay. Indeed, as predicted by the New Theory of Disuse (Bjork & Bjork, 1992), items with lower initial retrieval strength should stand to benefit more from being recalled in terms of facilitating subsequent accessibility than items with higher initial retrieval strength, thus potentially reducing or even reversing the effect of RIF observed on a subsequent final test.

Importantly, the present design cannot distinguish whether attenuation reflects the passage of time, the additional retrieval opportunity provided by the first test, or an interaction between these factors. Accordingly, the present findings should be interpreted as demonstrating that RIF is diminished across successive test assessments, rather than concluding that repeated testing itself caused attenuation of the effect. Future studies independently manipulating retention interval and the presence or absence of an initial final test assessment will be necessary to distinguish whether the recovery of Rp− items reflect spontaneous dissipation of inhibition, changes in context or stimulus conditions, or other changes in retrieval dynamics.

### Failed replication of exercise-related preservation of RIF

The principal contribution of the present investigation is not simply that the preservation effect observed in Experiment 3 of Loprinzi and Storm (2023) was not replicated. Rather, by employing a substantially larger sample and systematically manipulating exercise timing, intervention duration, and standing control conditions, the present study provides a more rigorous test of the robustness and generalizability of the previously observed effect. Although the present design cannot identify the precise source of the discrepancy between studies, it narrows the range of plausible theoretical explanations by demonstrating that systematic manipulation of several candidate mechanisms—including exercise timing, intervention duration, and standing control conditions—did not substantially alter the temporal evolution of RIF.

More broadly, the present findings underscore the importance of high-powered replication studies for cumulative psychological science. Replication studies that extend previous work while systematically evaluating competing mechanistic explanations provide critical opportunities to refine theoretical models, establish boundary conditions, and generate more precise estimates of effect magnitude. In this respect, the present investigation advances understanding of both RIF and exercise-cognition interactions by suggesting that the cognitive and retrieval processes underlying RIF may be comparatively resistant to acute physiological manipulation, thereby constraining theoretical accounts proposing generalized exercise-related enhancement of executive control during memory retrieval.

One important difference between the studies is the use of standing control conditions in the present experiment. Whereas Experiment 3 of Loprinzi and Storm (2023) contrasted exercise with seated control, the present design controlled for posture, alertness, and environmental engagement. It is therefore possible that the exercise effect observed by Loprinzi and Storm partially reflected differences between active and passive states rather than exercise-specific physiological mechanisms.

Additionally, the expanded design introduced longer total delays and additional exposure to retrieval, particularly in the dual-phase conditions. These factors may have overwhelmed any short-lived inhibitory modulation induced by exercise. If exercise effects on inhibition are temporally constrained, they may be masked when repeated retrieval allows suppressed items to recover.

### Exercise-related hypermnesia

Despite the absence of statistically significant exercise effects on RIF, we observed a descriptive pattern in which recall gains across repeated testing were sometimes numerically greater in the exercise conditions than in the standing-only control conditions. However, planned contrast comparing the three exercise groups with the two control groups did not provide statistical support for this interpretation. Accordingly, these data do not offer clear evidence for exercise-related hypermnesia. At most, they suggest a possibility that could be evaluated more directly in future work.

### Implications for theories of RIF

The present findings contribute to the RIF literature by demonstrating that although the classic RIF effect was robustly observed on the initial test, its magnitude diminished on the subsequent final test regardless of the intervening condition. This attenuation has important implications for theoretical accounts of RIF. Consistent with inhibitory accounts of RIF, selective retrieval may temporarily suppress competing representations, but the present results suggest that the accessibility of these representations can recover over time and across additional retrieval opportunities. Alternatively, competition-based accounts predict that repeated assessment alters the relative strength of competing retrieval pathways, or their likelihood of causing competition, thereby reducing the magnitude of the RIF effect.

More broadly, these findings refine current theories of RIF by identifying important boundary conditions governing the influence of acute exercise on retrieval processes. Identifying the mnemonic processes that are - and are not - susceptible to acute physiological modulation represents an important step toward developing more comprehensive theoretical models of both RIF and exercise-cognition interactions.

### Limitations

The present study was well powered to detect small-to-moderate interaction effects, although extremely small interaction effects cannot be ruled out. Physiological mechanisms were inferred rather than directly measured, and repeated final tests may have encouraged strategy shifts. Moreover, the rigorous standing control may have reduced contrast between conditions, making exercise effects more difficult to detect.

### Future directions

Future work should examine whether exercise influences RIF under conditions of greater interference, longer retention intervals, or increased retrieval difficulty. Combining acute exercise with measures of cardiorespiratory fitness or neurophysiological markers may clarify boundary conditions under which exercise modulates forgetting.

## Conclusion

Collectively, the present findings indicate that acute exercise did not reliably preserve RIF under the conditions examined, thereby refining theoretical boundary conditions for exercise-related modulation of RIF and providing a more precise estimate of the robustness of this effect.

## Data Availability

Data is available upon request.

## Footnotes

1 The higher overall recall observed for Nrp- relative to Nrp+ items reflects the intentional use of high-frequency versus low-frequency category exemplars rather than any consequence of the experimental manipulation, consistent with the methodology employed in previous retrieval-induced forgetting investigations.
